# Complete Genome Sequence of *Klebsiella pneumoniae* YH43

**DOI:** 10.1128/genomeA.00242-16

**Published:** 2016-04-14

**Authors:** Tadayuki Iwase, Yoshitoshi Ogura, Tetsuya Hayashi, Yoshimitsu Mizunoe

**Affiliations:** a Department of Bacteriology, The Jikei University School of Medicine, Minato-ku, Tokyo, Japan; b Department of Bacteriology, Faculty of Medical Sciences, Kyushu University, Higashi-ku, Fukuoka, Japan

## Abstract

We report here the complete genome sequence of *Klebsiella pneumoniae* strain YH43, isolated from sweet potato. The genome consists of a single circular chromosome of 5,520,319 bp in length. It carries 8 copies of rRNA operons, 86 tRNA genes, 5,154 protein-coding genes, and the *nif* gene cluster for nitrogen fixation.

## GENOME ANNOUNCEMENT

The genus *Klebsiella* belongs to the family *Enterobacteriaceae*. Bacteria belonging to the genus *Klebsiella* are nonmotile, rod-shaped, Gram negative, and produce polysaccharide capsule. This genus consists of 7 species, including *Klebsiella pneumoniae*. *K. pneumoniae* is a free-living bacterium, which can be found in soil, water, and a wide range of plants and animals, including humans. While *K. pneumoniae* is the medically most important species in this genus, the organism is also known as a nitrogen-fixing bacterium in agriculture (1–6). Recently, we isolated a *K. pneumoniae* strain (YH43) from sweet potato (*Ipomoea batatas*) with nitrogen-fixing ability. To gain the genetic basis for analyzing the characteristics of this bacterium, we have first determined its complete genome sequence.

The genome sequence of strain YH43 was determined using the 454 GS FLX Titanium system (Roche). A total of 524,416 single-end reads (218 Mb) and 142,440 8-kb paired-end reads (46 Mb) were assembled with the GS Assembler software version 2.6 into a single scaffold containing 58 gaps. The gaps were closed by *in silico* analysis using the GenoFinisher software (7) (http://www.ige.tohoku.ac.jp/joho/gf_e/), and gap-spanning PCR products were sequenced using an ABI3130xl DNA sequencer (Applied Biosystems). To correct sequence errors, a paired-end library of the YH43 genome was constructed using the TruSeq DNA sample prep kit (Illumina) and sequenced by the Illumina MiSeq platform (2 × 150 bp). By mapping the obtained 2,085,021 reads (626 Mb) to the initial genome sequence using BWA (8) and SAMtools (9), a total of 38 insertions/deletions were corrected. The gene identification and annotation were conducted using the Microbial Genome Annotation Pipeline (MiGAP) (http://www.migap.org).

The complete sequence of the YH43 genome consists of a single circular chromosome of 5,520,319 bp in length. No plasmid was identified. The chromosome contains 8 copies of the rRNA operons, 86 tRNA genes, and 5,154 protein-coding genes. It is known that one-third of *Klebsiella* isolates have nitrogen-fixing ability (10), which one of the model systems for studying nitrogen fixation (5). The *nif* gene cluster consisted of 20 genes that were identified on the YH43 chromosome. The number and orientation of genes were consistent with a previous report on strain *K. pneumoniae* 342 (11), which was isolated from the stems of *Zea mays* L. cv. CIMMYT 342 (12), suggesting a well-conserved genetic organization of the *nif* gene cluster among *K. pneumoniae* strains.

### Nucleotide sequence accession number.

This whole-genome shotgun project has been deposited at DDBJ under the accession no. AP014950.

## References

[1] Postgate JR 1970 Biological nitrogen fixation. Nature 226:25–27. doi:10.1038/226025a0. 4985009

[2] Zumft WG, Mortenson LE 1975 The nitrogen-fixing complex of bacteria. Biochim Biophys Acta 416:1–52. doi:10.1016/0304-4173(75)90012-9. 164247

[3] Andersen K, Shanmugam KT, Valentine RC 1977 Nitrogen fixation (NIF) regulatory mutants of *Klebsiella*: determination of the energy cost of N_2_ fixation *in vivo*. Basic Life Sci 9:95–110. 336030 10.1007/978-1-4684-0880-5_9

[4] Roberts GP, Brill WJ 1981 Genetics and regulation of nitrogen fixation. Annu Rev Microbiol 35:207–235. doi:10.1146/annurev.mi.35.100181.001231. 7027900

[5] Drummond MH 1984 The nitrogen fixation genes of *Klebsiella pneumoniae*: a model system. Microbiol Sci 1:29–33. 6444092

[6] Gussin GN, Ronson CW, Ausubel FM 1986 Regulation of nitrogen fixation genes. Annu Rev Genet 20:567–591. doi:10.1146/annurev.ge.20.120186.003031. 3545064

[7] Ohtsubo Y, Maruyama F, Mitsui H, Nagata Y, Tsuda M 2012 Complete genome sequence of *Acidovorax* sp. strain KKS102, a polychlorinated-biphenyl degrader. J Bacteriol 194:6970–6971. doi:10.1128/JB.01848-12. 23209225 PMC3510582

[8] Li H, Durbin R 2009 Fast and accurate short read alignment with Burrows–Wheeler transform. Bioinformatics 25:1754–1760. doi:10.1093/bioinformatics/btp324. 19451168 PMC2705234

[9] Li H, Handsaker B, Wysoker A, Fennell T, Ruan J, Homer N, Marth G, Abecasis G, Durbin R, 1000 Genome Project Data Processing Subgroup 2009 The sequence alignment/map format and SAMtools. Bioinformatics 25:2078–2079. doi:10.1093/bioinformatics/btp352. 19505943 PMC2723002

[10] Mahl MC, Wilson PW, Fife MA, Ewing WH 1965 Nitrogen fixation by members of the tribe *Klebsielleae*. J Bacteriol 89:1482–1487. 14291584 10.1128/jb.89.6.1482-1487.1965 PMC277680

[11] Fouts DE, Tyler HL, DeBoy RT, Daugherty S, Ren Q, Badger JH, Durkin AS, Huot H, Shrivastava S, Kothari S, Dodson RJ, Mohamoud Y, Khouri H, Roesch LF, Krogfelt KA, Struve C, Triplett EW, Methé BA 2008 Complete genome sequence of the N_2_-fixing broad host range endophyte *Klebsiella pneumoniae* 342 and virulence predictions verified in mice. PLoS Genet 4:e1000141. doi:10.1371/journal.pgen.1000141. 18654632 PMC2453333

[12] Chelius MK, Triplett EW 2000 Immunolocalization of dinitrogenase reductase produced by *Klebsiella pneumoniae* in association with *Zea mays* L. Appl Environ Microbiol 66:783–787. doi:10.1128/AEM.66.2.783-787.2000. 10653751 PMC91896

